# Integrative Genetic Characterization and Phenotype Correlations in Pheochromocytoma and Paraganglioma Tumours

**DOI:** 10.1371/journal.pone.0086756

**Published:** 2014-01-22

**Authors:** Joakim Crona, Margareta Nordling, Rajani Maharjan, Dan Granberg, Peter Stålberg, Per Hellman, Peyman Björklund

**Affiliations:** 1 Department of Surgical Sciences, Uppsala University, Uppsala, Sweden; 2 Department of Medical Sciences, Uppsala University, Uppsala, Sweden; 3 Department of Clinical Genetics, Sahlgrenska University Hospital, Göteborg, Sweden; Cleveland Clinic Lerner Research Institute, United States of America

## Abstract

**Background:**

About 60% of Pheochromocytoma (PCC) and Paraganglioma (PGL) patients have either germline or somatic mutations in one of the 12 proposed disease causing genes; *SDHA*, *SDHB*, *SDHC*, *SDHD*, *SDHAF2*, *VHL*, *EPAS1*, *RET*, *NF1*, *TMEM127, MAX* and *H-RAS*. Selective screening for germline mutations is routinely performed in clinical management of these diseases. Testing for somatic alterations is not performed on a regular basis because of limitations in interpreting the results.

**Aim:**

The purpose of the study was to investigate genetic events and phenotype correlations in a large cohort of PCC and PGL tumours.

**Methods:**

A total of 101 tumours from 89 patients with PCC and PGL were re-sequenced for a panel of 10 disease causing genes using automated Sanger sequencing. Selected samples were analysed with Multiplex Ligation-dependent Probe Amplification and/or SNParray.

**Results:**

Pathogenic genetic variants were found in tumours from 33 individual patients (37%), 14 (16%) were discovered in constitutional DNA and 16 (18%) were confirmed as somatic. Loss of heterozygosity (LOH) was observed in 1/1 *SDHB*, 11/11 *VHL* and 3/3 NF1-associated tumours. In patients with somatic mutations there were no recurrences in contrast to carriers of germline mutations (*P* = 0.022). *SDHx*/*VHL*/*EPAS1* associated cases had higher norepinephrine output (*P* = 0.03) and lower epinephrine output (*P*<0.001) compared to *RET*/*NF1*/*H-RAS* cases.

**Conclusion:**

Somatic mutations are frequent events in PCC and PGL tumours. Tumour genotype may be further investigated as prognostic factors in these diseases. Growing evidence suggest that analysis of tumour DNA could have an impact on the management of these patients.

## Introduction

Pheochromocytoma (PCC) and paraganglioma (PGL) are rare neural crest-derived tumours arising in the adrenal medulla (PCC) or autonomic ganglia (PGL). A majority of patients present with a focal tumour lesion and may be cured with R0 resection [1]–[3]. However, even following an apparently successful surgical resection, there may be a risk of local or metastatic recurrence that motivates a long follow up period [2], [4], [5]. Age, familial disease and tumour size correlate to increased risk of malignancy and recurrence [2] and histologic criteria may also aid in predicting risk for malignant disease [6], [7]. Translational studies show that approximately 60% of PCC and PGL cases have either germline or somatic mutations in one of 13 suggested disease causing loci; *SDH* subunits *A*, *B*, *C* and *D*, *SDHAF2*, *VHL*, *EPAS1*, *RET*, *NF1*, *TMEM127*, *MAX* and *H-RAS* [8]–[20]. In the clinical setting, genetic screening of these genes by fragment prioritization of germline DNA is regarded as golden standard of care, and may have a substantial impact on patient management [21], [22]. Depending on the affected gene, the risk of local recurrence and/or metastatic disease can be estimated and guide in the selection of appropriate preventive measures [22]. Screening for tumour specific genetic events is not recommended in clinical practice due to a lack of genotype-phenotype correlations that could justify the necessary resource allocation [8], [23]. However, as most genetic screening studies of PCC and PGL tumours have been performed on small cohorts, we hypothesized that a comprehensive genetic screening in a large clinically annotated cohort could be informative. The aim of this study was, to describe the genetic landscape of PCC and PGL tumours and to correlate tumour genotype with patient characteristics and disease outcome.

## Patients and Methods

### Patients

This is a single centre, retrospective study of 101 tumour samples from 89 patients with PCC and PGL treated at the Department of Surgery, Uppsala university hospital, Sweden. Selected patients were previously screened for mutations in *H-RAS* describing somatic genetic variants (*n = *4) and *MAX* describing no pathogenic genetic variant [20], [24]. Fourteen patients were clinically diagnosed with hereditary syndromes; familial paraganglioma type 4 (PGL4; *n* = 2), Von Hippel Lindau syndrome (VHL; *n* = 4) and Multiple Endocrine Neoplasia type 2 (MEN2; *n* = 8) by certified genetic testing laboratories. Four additional patients had been diagnosed with Neurofibromatosis type 1 (NF1) by presence of clinical criteria. DNA samples from 195 healthy and unrelated individuals were utilized as a control to determine frequency of germline variants with unknown significance (VUS) in Swedish population.

### Ethical Statement

Ethical approval was obtained from the regional ethics committee in Uppsala as well as written informed consent from the individual patients. All patients were above 18 years of age at the time of inclusion.

### Clinical Data

Age at diagnosis was set at the time for radiological diagnosis. Tumour size was calculated as the mean of two diameters. Cases were classified as metastatic by the presence of invasion into nonchromaffin organs determined by histological examination or radiological/molecular imaging. Preoperative urinary catecholamines measurements analysed in a clinical setting were included for evaluation. Norepinephrine assays had been performed with two different reference intervals, <350 nmol/24 h and <400 nmol/24 h depending on utilized assays. Reference intervals for urinary epinephrine was <90 nmol/24 h, for plasma normetanephrine <0,6 nmol/L and for plasma metanephrine <0,3 nmol/L.

### DNA Extraction & Sequencing

DNA was extracted from cryosections of tumour samples, peripheral blood and/or normal tissue, using DNeasy Blood & Tissue Kit (Qiagen, Hilden, Germany) as previously described [25]. Cryosections from included tumours were analysed for tissue morphology and selected samples were macrodissected in order to reduce contamination of normal cells. Using a phenotype guided fragment prioritization approach [1], [26], exons and intron-exon boundaries of *SDHB* (NM_003000.2), *SDHC* (NM_003001.3), *SDHD* (NM_003002.2), *SDHAF2* (NM_017841.2), *VHL* (NM_000551.3), *EPAS1* (exons 9 and 12, NM_001430.4), *RET* (exons 10–11 and 13–16, NM_020975.4), *TMEM127* (NM_017849.3), *MAX* (NM_002382.3) and *H-RAS* (exons 2 and 3, NM_176795.3) were amplified by PCR and sequenced using automated Sanger sequencing (Beckman Coulter Genomics, Takeley, UK). In patients with pathogenic germline variants, all exons and intron-exon boundaries of the affected gene were sequenced in order to investigate a potentially inactivating variant on the 2^nd^ allele. Ultimately, patients without detected pathogenic mutations in this study had been screened for all ten above mentioned genes. Primer sequences can be obtained by request.

### Mutational Analysis

Chromatograms generated by Sanger Sequencing were reviewed using CLC genomics workbench 5.5 (CLC bio, Aarhus, Denmark). Genetic variants were annotated for overlapping information available in public databases; Catalogue of Somatic Mutations in Cancer (COSMIC) [27], the Single Nucleotide Polymorphism database (dbSNP), Human Genome Mutation Database (HGMD public) [28] and Leiden Open source Variation Database (LOVD). *In silico* analysis was performed using Sorting Intolerant From Tolerant (SIFT) [29] and Polymorphism Phenotyping v2 (Polyphen-2) [30].

### Multiplex Ligation-dependent Probe Amplification

Inclusion criteria for analysis were absence of a pathogenic germline variant. Only DNA extracted from blood/normal tissue was selected for Multiplex ligation-dependent probe amplification (MLPA). Included samples were analysed with SALSA MLPA P226 SDH and P016-B2 VHL probe mixes (MRC-Holland, Amsterdam, Netherlands) that have coverage of the *SDHA*, *SDHB*, *SDHC*, *SDHD*, *SDHAF2* and *VHL* loci. Reactions were carried out as previously described [31] and an ABI3130xl Genetic Analyzer (Life Technologies, Carlsbad, CA) was used for fragment separation. The MLPA data were analyzed using GeneMapper 4.0 genotyping software (Applied Biosystem) and SeqPilot version 3.3.2 (JSI medical systems GmBH, Kippenheim, Germany). The experiments were carried out at a laboratory certified for clinical use and analysed by an experienced clinical investigator (MN).

### Single Nucleotide Polymorphism Array

Inclusion criteria were presence of pathogenic or unknown variants in *SDHB*, *SDHC, VHL* or clinical criteria of NF1. Tumour DNA from the selected samples were subjected to SNParray analysis; using Illumina Omni1-Quad or Omni2,5-Quad chips (Illumina Inc, CA, USA), containing 1,140,419 and 2,379,855 probes respectively. Hybridization and sequencing was performed by university core facilities, SNP&SEQ Technology Platform in Uppsala, Sweden (http://molmed.medsci.uu.se/SNPSEQTechnologyPlatform/). Generated data was imported into the Illumina BeadStudio (Illumina, CA, USA) software and analysed using Nexus Copy Number Variation 7.0 Build 7887 (Biodiscovery Inc, CA, USA) to detect copy number variation and allelic imbalance using default thresholds The fraction of DNA derived from tumour cells were estimated by analysing the B-allele frequency in regions showing copy number alterations [32]. Samples with tumour cell purity <70% were analysed with adjusted settings.

### Statistics

SPSS 19 (IBM, Armonk NY, US) was used for statistical calculations. Chi^2^ test was performed for analysis of nominal variables. As age at diagnosis, tumour size and catecholamine output were not normally distributed, hence a non-parametric test (Mann-Whitney U test) was selected for analysis of these scaled values. Patients with variants of unknown significance (VUS) were classified as wild type. Multiple regression test of phenotype correlation to carrier status and genotypes were not possible to perform due to the low number of observations. P-values <0.05 were considered as significant and <0.1 as borderline significant.

## Results

Patient characteristics are described in table 1. The median age at diagnosis was 49 years (range 15–85) and there were 35 males and 53 females. Eighty patients had adrenal PCC (31 left adrenal, 37 right adrenal, 10 bilateral), eight had thoracoabdominal PGLs and there were one head and neck PGL. The median tumour size was 55 mm (range 8–170). There were wide discrepancies in catecholamine output; urinary norepinephrine range 112–19130 nmol/24 h (ref <400/<350 nmol/24 h), urinary epinephrine range 19–33322 nmol/24 h (ref <90 nmol/24 h), plasma normetanephrine range 1–37 nmol/L (ref <0,6 nmol/L), and plasma metanephrine range 0–140 nmol/L (ref <0,3 nmol/L). There were 13 patients with recurrent disease, eight of whom recurred with distant metastases and five with local recurrences. One additional patient had distant metastases at the time of diagnosis. The median follow up time was 106 months (range 0–714 months).

**Table 1:** Clinical characteristics and carrier status.

		Cohort (*n* = 89)		No discovered mutations (*n* = 52)*		Germline variants (*n* = 18)**		Somatic variants (*n* = 16)		Germline and no mutation	Somatic and no mutation	Germline and Somatic mutation
			Range		Range		Range		Range	*P*		
Median age, years		49,5	15–85	53	22–85	29,5	15–66	48	25–81	0.002*^§^*	0.622*^§^*	*0.025^§^*
Gender, Male/Female		35/53		19/32		7/11		8/8		0.902*^§§^*	0.365*^§§^*	*0.515^§§^*
Tumour specifications												
Median size, mm		55	8–170	60	20–140	51 (n = 14)	20–170	45	8–100	0.166*^§^*	0.305*^§^*	*0.755^§^*
Localization, left adrenal/right adrenal		31/37		22/21		3/5		5/9		0.478*^§§^*	0.315*^§§^*	*0.933^§§^*
Adrenal/extra adrenal tumour		80/9		47/5		16/2		14/2		0.855*^§§^*	0.740*^§§^*	*0.900^§§^*
Multifocal tumours		10		1		9		0		<0.001*^§§^*	0.561*^§§^*	*<0.001^§§^*
Biochemistry												
Median Urine Norepinephrine, nmol/24 h		1687	112–19130	2181,5	230–19130	836	112–4545	2439	225–14244	0.033*^§^*	0.962*^§^*	*0.049^§^*
Median Urine Epinephrine, nmol/24 h		189	19–33322	247	19–33322	195	27–641	96	25–11664	0.2*^§^*	0.541*^§^*	*0.622^§^*
Median Plasma Normetanephrine, nmol/L		6,4	1–37	7,1	4–16	1,4	1–2	11,6	1–37	0.004*^§^*	0.640*^§^*	*0.136^§^*
Median Plasma Metaepinephrine, nmol/L		0,5	0–140	1,65	0–34	0,45	0–1	1,05	0–140	0.579*^§^*	0.961*^§^*	*0.391^§^*
Recurrent disease		13		8		5		0		0.376*^§§^*	0.070*^§§^*	*0.022^§§^*
Metastatic disease***		9		7		2		0		0.797*^§§^*	0.121*^§§^*	*0.169^§§^*

Statistical correlation of clinical variables and carriers status. Multifocal tumours was defined as bilateral pheochromocytoma or multiple paraganglioma tumours.

* Patients with no available germline DNA were excluded.

** Included 14 germline variants and 4 patients with clinical criteria of Neurofibromatosis type 1.

*** Nine patients had metastatic disease, one of these had metastatic disease at the time of diagnosis and the remaining had metastatic recurrences later on in the disease course. §Mann-Whitney U Test and §§Chi Square test.

### Genetic Screening

**Figure 1:**
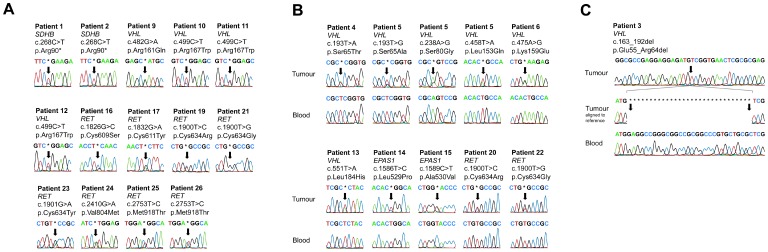
Chromatograms exported from CLC Genomics Workbench 5.5 displaying (A) Pathogenic genetic variants available in constitutional DNA, (B) confirmed somatic variants and (C) 30 base pair somatic deletion in *VHL*.

A total of 33 patients (37%) had a pathogenic genetic variant in included disease causing loci. We could confirm 14 of these mutations in constitutional DNA (Figure 1a) and 16 were confirmed as somatic by absence in constitutional DNA (Figures 1b and c). There was no constitutional DNA available for three of the patients with pathogenic mutations. All genetic variants previously clinically diagnosed by the Department of Clinical Genetics (*n = *14) were verified in the present study. There were four additional patients with clinical criteria of Neurofibromatosis type 1 but with no genetic testing. Cases with discovered mutations and corresponding clinical characteristics are presented in Table 2.

**Table 2:** Pathogenic and unknown genetic varaints and their corresponding patient characteristics.

Patient no.	Diagnosis	Gender	Age at diagnosis	Hereditary	Syndrome criteria	Size (mm)	Unilateral/multiple	Recurrent	Metastatic	Gene	Exon	Somatic/Germline	cDNA	Amino acid substitution	Concluded Pathogenicity
1	TA PGL	M	15	+	PGL4	-	Multiple	+	−	*SDHB*	3	Germline	c.268C>T	p.Arg90*	Pathogenic
2	TA PGL	F	26	+	PGL4	-	Multiple	+	−	*SDHB*	3	Germline	c.268C>T	p.Arg90*	Pathogenic
3	PCC	M	76	−	−	70	Uni	−	−	*VHL*	1	Somatic	c.163_192del	p.Glu55_Arg64del	Pathogenic
4	PCC	M	58	−	−	90	Uni	−	−	*VHL*	1	Somatic	c.193T>A	p.Ser65Thr	Pathogenic
5	PCC	F	49	−	−	25	Uni	−	−	*VHL*	1	Somatic	c.193T>G	p.Ser65Ala	Pathogenic
6	PCC	F	25	−	−	25	Uni	−	−	*VHL*	1	Somatic	c.238A>G	p.Ser80Gly	Pathogenic
7	PCC	F	47	−	−	8	Uni	−	−	*VHL*	2	Somatic	c.458T>A	p.Leu153Gln	Pathogenic
8	PCC	M	47	−	−	100	Uni	−	−	*VHL*	3	Somatic	c.475A>G	p.Lys159Glu	Pathogenic
9	PCC	F	66	NA	VHL	65/60	Multiple	−	−	*VHL*	3	Germline	c.482G>A	p.Arg161Gln	Pathogenic
10	PCC	F	25	+	VHL	60/40	Multiple	−	−	*VHL*	3	Germline	c.499C>T	p.Arg167Trp	Pathogenic
11	PCC	M	21	+	VHL	40/30	Multiple	−	−	*VHL*	3	Germline	c.499C>T	p.Arg167Trp	Pathogenic
12	PCC	F	25	+	VHL	30	Uni	−	−	*VHL*	3	Germline	c.499C>T	p.Arg167Trp	Pathogenic
13	PCC	F	31	−	−	40	Uni	−	−	*VHL*	3	Somatic	c.551T>A	p.Leu184His	Pathogenic
14	TA PGL	F	64	−	−	20	Uni	−	−	*EPAS1*	12	Somatic	c.1586T>C	p.Leu529Pro	Pathogenic
15	PCC	F	81	−	−	45	Uni	−	−	*EPAS1*	12	Somatic	c.1589C>T	p.Ala530Val	Pathogenic
16	PCC	F	27	+	MEN2A	100	Uni	−	−	*RET*	10	Germline	c.1826G>C	p.Cys609Ser	Pathogenic
17	PCC	F	57	+	MEN2A	23	Multiple	+	−	*RET*	10	Germline	c.1832G>A	p.Cys611Tyr	Pathogenic
18	PCC	F	61	−	−	15	Uni	−	−	*RET*	11	NA	c.1891G>T	p.Asp631Tyr	Pathogenic
19	PCC	F	29	+	MEN2A	30/47	Multiple	−	−	*RET*	11	Germline	c.1900T>C	p.Cys634Arg	Pathogenic
20	PCC	F	47	−	−	80	Uni	−	−	*RET*	11	Somatic	c.1900T>C	p.Cys634Arg	Pathogenic
21	PCC	M	29	+	MEN2A	20/NA	Multiple	+	−	*RET*	11	Germline	c.1900T>G	p.Cys634Gly	Pathogenic
22	PCC	F	57	−	−	60	Uni	−	−	*RET*	11	Somatic	c.1900T>G	p.Cys634Gly	Pathogenic
23	PCC	F	30	+	MEN2A	20/NA	Multiple	−	−	*RET*	11	Germline	c.1901G>A	p. Cys634Tyr	Pathogenic
24	PCC	M	65	-	MEN2A	95	Uni	+	+	*RET*	14	Germline	c.2410G>A	p.Val804Met	Pathogenic
25	PCC	F	18	−	MEN2B	25	Uni	+	−	*RET*	16	Germline	c.2753T>C	p.Met918Thr	Pathogenic
26	PCC	M	34	−	MEN2B	60/60	Multiple	−	−	*RET*	16	Germline	c.2753T>C	p.Met918Thr	Pathogenic
27	PCC	M	45	−	−	30	Uni	NA	NA	*RET*	16	NA	c.2753T>C	p.Met918Thr	Pathogenic
28	PCC	F	31	−	−	55	Uni	−	−	*RET*	16	NA	c.2753T>C	p.Met918Thr	Pathogenic
29	PCC	M	54	−	−	45	Uni	−	−	*H-RAS*	2	Somatic	c.37G>C	p.Gly13Arg	Pathogenic
30	PCC	M	76	−	−	76	Uni	−	−	*H-RAS*	3	Somatic	c.181C>A	p.Gln61Lys	Pathogenic
31	PCC	M	36	−	−	30	Uni	−	−	*H-RAS*	3	Somatic	c.181C>A	p.Gln61Lys	Pathogenic
32	TA PGL	M	31	−	−	100	Uni	−	−	*H-RAS*	3	Somatic	c.182A>G	p.Gln61Arg	Pathogenic
33	PCC	M	45	−	−	100	Uni	−	−	*H-RAS*	3	Somatic	c.182A>G	p.Gln61Arg	Pathogenic
34	PCC	F	61	−	−	25	Uni	−	−	*SDHC*	5	Germline	c.328C>T	p.Pro110Ser	Unknown
35	PCC	M	55	−	−	65	Uni	−	−	*SDHC*	6	Germline	c.490A>T	p.Met164Val	Unknown
36	TA PGL	M	28	−	−	20	Uni	−	−	*VHL*	3	Germline	c.548C>T	p.Ser183Leu	Unknown
37	PCC	F	27	−	−	50	Uni	−	−	*RET*	13	Germline	c.2372A>T	p.Tyr791Phe	Unknown

PCC; Pheochromocytoma, TA PGL; Thoracoabdominal Paraganglioma, NA; Not Available, F; Female, M; Male, MEN2; Multiple Endocrine Neoplasia type 2, PGL4; Familial Paraganglioma type 4, VHL; Von Hippel Lindau, Uni; Unilateral or focal tumour lesion.

Two related patients (mother and son) presented with multiple abdominal PGL. Both had several local recurrences that were not classified as metastatic lesions. Re-sequencing revealed a pathogenic nonsense mutation in *SDHB*; c.268C>T, p.Arg90* [13] that was present in DNA from blood.

There were 12 cases with pathogenic mutations in the *VHL* gene. Patient number 9 with bilateral PCC (index case) had a pathogenic germline missense mutation in *VHL*; c.482G>A p.Arg161Gln. A family comprising of three siblings with bilateral or unilateral PCC had pathogenic germline missense mutation in *VHL*; c.499C>T, p.Arg167Trp [33]. Both p.Arg161Gln and p.Arg167Trp had previously been reported as pathogenic [33]. Seven patients had somatic mutations in *VHL*. There were six unique SNVs; c.193T>G, p.Ser65Ala; c.193T>A, p.Ser65Thr; c.238A>G, p.Ser80Gly; c.458T>A, p.Leu153Gln; c.475A>G, p.Lys159Glu and c.551T>A, p.Leu184His in one patient each. Patient number 4 had a 30 base pair deletion c.163_192del, p.Glu55_Arg64del that was absent in DNA from peripheral blood. All carriers of somatic *VHL* mutations had unilateral PCC, sporadic disease presentation and there were no apparent signs or symptoms of VHL syndrome.

Two patients had mutations in *EPAS1* which were absent in DNA from their blood; one c.1586T>C, p.Leu529Pro and one c.1589C>T, p.Ala530Val. These mutations are previously described as pathogenic [16], [34]. Both patients had sporadic disease presentation. Patient 16 had borderline polycytemia with a haemoglobin level of 150 g/L (reference interval 120–150 g/L).

There were 13 patients with pathogenic mutations in *RET*. Eight had germline pathogenic mutations and clinical characteristics of MEN2 syndrome; c.1826G>C, p.Cys609Ser; c.1832G>A, p.Cys611Tyr; c.1900T>C, p.Cys634Arg; c.1900T>G, p.Cys634Gly; c.1901G>A, p. Cys634Tyr; c.2410G>A, p.Val804Met in one patient each and c.2753T>C, p.Met918Thr in tow different patients. Two patients with unilateral PCC and sporadic disease presentation had somatic mutation in *RET*; c.1900T>G, p.Cys634Gly and c.1900T>C, p.Cys634Arg. For three of the patients with SNVs in *RET* there were no constitutional DNA available; c.1891G>T, p.Asp631Tyr in one patient and c.2753T>C, p.Met918Thr in two patients. All three cases had sporadic disease presentation and there were no signs or symptoms suggesting MEN2 syndrome. All these *RET* mutations are described as pathogenic in the literature [8], [33].

Four patients had previously been described with somatic *H-RAS* mutations [20]. One additional somatic mutation in *H-RAS;* c.181C>A, p.Gln61Lys; was detected in a male patient that had sporadic disease presentation.

### Variants of Unknown Significance

Four patients had germline variants of unknown significance (VUS). There were two VUS in *SDHC*; c.328C>T, Pro110Ser [35] and c.490A>T, Met164Leu in two different patients with unilateral PCC and sporadic disease presentation. Both mutations were available in constitutional DNA. *Succinate dehydrogenase subunit C* Met164Leu has been reported to have impact in functional models but was classified as benign in vivo [36]. The pathogenicity of Pro110Ser has not been investigated in detail [35]. *Succinate dehydrogenase subunit C* Codon 110 is conserved among vertebrate orthologs whereas codon 164 is not a conserved residue. *In silico* analysis determined the variants as benign, SIFT (0,93 and 0,96) and Polyphen2 (0,231 and 0,0).

Patient 36, a 28 year old male, was referred to the local hospital due to hypertension, headache and multiple episodes of syncope. Urinary norepinephrine was elevated, 1752 nmol/24 h (<350), but epinephrine was within reference margins 37 nmol/24 h (<90). A computed tomography revealed an abdominal mass located in close proximity to the left renal artery and vein. The lesion was surgically resected and the pathology report showed a PGL, 20 mm in size with a Ki67 index of <1%. Genetic screening of *RET* as well as *Succinate Dehydrogenase* subunits *B* and *D* was normal. The proband remains free of recurrence 21 months following the initial diagnosis. There were no apparent signs or symptoms of von Hippel Lindau syndrome. Sanger sequencing revealed a single nucleotide polymorphism in *VHL*; c.548C>T, p.Ser183Leu. Multiplex ligation-dependent probe amplification of constitutional DNA did not show any pathological imbalances. In tumour tissue, loss of heterozogosity and copy number loss was observed on the whole arm of chromosome 3p by Omni-1-quad SNP array (Illumina, CA, USA).*VHL*; c.548C>T, p.Ser183Leu has been classified as pathogenic in a functional model but the individual contribution of the allele to patient phenotype is not fully described [37]. Screening of 190 healthy individuals revealed homozygous C allele in all cases. Codon 183 is conserved among mammalian orthologs and *in silico* analysis determined the variant as probably pathogenic: SIFT (0,18) and Polyphen2 (1,0).

Patient 37 was diagnosed with a unilateral PCC at 27 years of age having apparently sporadic presentation. Re-sequencing revealed a *RET* mutation c.2372A>T, p.Tyr791Phe that was found in constitutional DNA. The pathogenicity of *RET* p.Tyr791Phe is disputed [38], [39].

There were no pathogenic variants discovered in *SDHAF2*, *TMEM127* and *MAX*.

### Structural Variations

**Figure 2:**
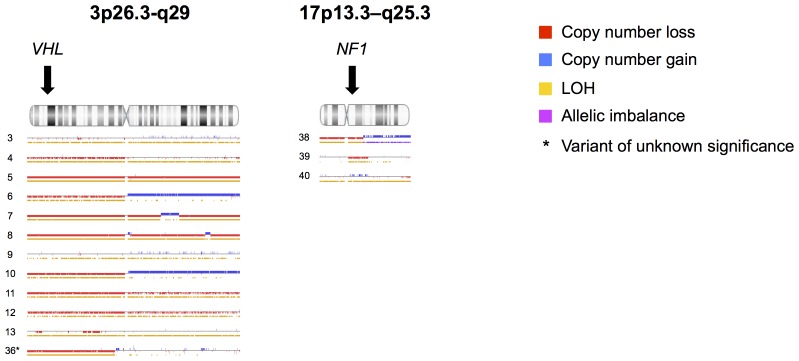
Detected copy number events from SNP array as displayed by Nexus copy number 7.0. Results are separated by chromosome and presented for each individual tumour with patient id at the left margin. Presented data constitute cases harbouring pathogenic genetic variants in *VHL* (*n* = 11) as well as patients with clinical criteria of NF1 (*n = *3). Data is also presented for patient 36 that harboured a *VHL* mutation of unknown significance. Colour annotation indicates copy number loss (red), copy number gain (blue), loss of heterozygosity (yellow) and allelic imbalance (magenta). Arrows indicates *VHL* (chromosome 3) and *NF1* (chromosome 17) loci. Loss of heterozygosity at *VHL* locus was detected in 11/11 tumours with pathogenic *VHL* mutations and in 3/3 tumours from patients with clinical criteria of NF1.

Multiplex ligation-dependent probe amplification analysis did not reveal any copy number gains or losses. Analysis of SNP array data showed loss of heterozygosity (LOH) located to the genomic coordinates of the respective gene in 1/1 *SDHB*, 11/11 *VHL* and 3/3 NF1-related tumours (table 3, figure 2). There were no LOH at coordinates corresponding to *SDHC* loci in tumours from patients with germline *SDHC* Pro110Ser and Met164Leu variants. Patient 36 with a germline *VHL p.*Ser183Leu had LOH at the *VHL* loci. Analysis failed due to corrupted data in two cases, one *SDHB* and one *NF1*-related tumour.

**Table 3:** Copy number variation and loss of heterozygosity by SNP array.

				Copy number variation		Loss of heterozogozity		
Patient no.	Gene	Amino acid substitution	Chromosome	Start position	End position	Start position	End position	Nexus 7.0 Quality score
3	*VHL*	p.Glu55_Arg64del	3	Not detected		1	194000000	0,018
4	*VHL*	p.Ser65Thr	3	1	85000000	1	194000000	0,046
5	*VHL*	p.Ser65Ala	3	1	194000000	1	194000000	0,016
6	*VHL*	p.Ser80Gly	3	1	85000000	1	85000000	0,017
7	*VHL*	p.Leu153Gln	3	1	120000000	1	120000000	0,074
8	*VHL*	p.Lys159Glu	3	1	194000000	1	194000000	0,028
9	*VHL*	p.Arg161Gln	3	Not detected		1	194000000	0,024
10	*VHL*	p.Arg167Trp	3	1	87000000	1	87000000	0,022
11	*VHL*	p.Arg167Trp	3	1	194000000	1	194000000	0,025
12	*VHL*	p.Arg167Trp	3	1	194000000	1	194000000	0,033
13	*VHL*	p.Leu184His	3	9000000	13000000	1	194000000	0,022
36	*VHL*	p.Ser183Leu	3	1	81000000	1	81000000	0,027
38	*NF1*	NA	17	1	39000000	1	39000000	0,026
39	*NF1*	NA	17	25000000	43000000	25000000	43000000	0,03
40	*NF1*	NA	17	Not detected		1	81000000	0,046

Tumours with loss of heterozygosity at loci of mutated tumour suppressor. Patients with diagnostic criteria of NF1 were considered as potential carriers of a pathogenic variant in the *NF1* gene. NA, Not Available.

### Statistical Correlation

Statistical analyses of carrier status and genotype correlations to phenotype are presented in Tables 1 and 4. Stratified into groups accordingly to mutation status, germline carriers had a age at diagnosis that were significantly lower (median 29,5 years) compared to those with somatic aberrations (median 48 years, *P = *0.025) as well as patients without known mutations (median 53 years, *P = *0.002). The frequency of mutifocal tumours were also different in germline carriers (53%) compared to patients with somatic carrier status (0%, *P*<0.001) as well as those without known mutations (2%, *P*<0.001).

**Table 4:** Clinical characteristics and genotype.

	No discovered mutations (*n* = 52)		Cluster 1 *SDHx*/*VHL*/*EPAS1 (*n* = 15)*		Cluster 2 *RET*/*NF1*/*H-RAS (*n* = 22)*		Cluster 1 and no mutation	Cluster 2 and no mutation	Cluster 1 and Cluster 2
				Range		Range	*P*		
Median age, years	53	22–85	47	15–81	45	18–76	*0.089^§^*	*0.036^§^*	*0.699^§^*
Gender, Male/Female	19/32		5/10		11/11		*0.781^§§^*	*0.310^§§^*	*0.315^§§^*
Tumour specifications									
Median size, mm	60	20–140	40	8–100	51	15–170	*0.086^§^*	*0.213^§^*	*0.658^§^*
Localization, left adrenal/right adrenal	22/21		1/8		8/8		*0.028^§§^*	*0.937^§§^*	*0.052^§§^*
Adrenal/extra adrenal tumour	47/5		12/3		21/1		*0.275^§§^*	*0.465^§§^*	*0.137^§§^*
Multifocal tumours	1		4		5		*<0.001^§§^*	*0.004^§§^*	*0.693^§§^*
Biochemistry									
Median Urine Norepinephrine, nmol/24 h	2181,5	230–19130	2439	495–14244	862	112–5189	*0.960^§^*	*0.018^§^*	*0.03^§^*
Median Urine Epinephrine, nmol/24 h	247	19–33322	58	25–96	520	39–11664	*0.002^§^*	*0.232^§^*	*<0.001^§^*
Median Plasma Normetanephrine, nmol/L	7,1	4–16	19,3	2–37	1,5	1–34	*1.0^§^*	*0.076^§^*	*0.327^§^*
Median Plasma Metaepinephrine, nmol/L	1,65	0–34	0,25	0,25	0,7	0–140	*0.304^§^*	*0.914^§^*	*0.073^§^*
Recurrent disease	8		2		3		*0.689^§§^*	*0.723^§§^*	*0.925^§§^*
Metastatic disease	7		0		2		*0.133^§§^*	*0.599^§§^*	*0.230^§§^*

Statistical analysis of correlation between clinical characteristics and genotype. § Mann-Whitney U Test, §§ Chi-square test, * Patients with clinical criteria of *NF1* defined as having germline carrier status.

No cases of recurrent disease were observed in somatic carriers (0%), in contrast to germline carriers (28%, *P = *0.022) and borderline significant compared to patients without discovered mutations (15%, *P = *0.07). Preoperative levels of urine norepinephrine were lower in patients with germline carrier status compared to somatic carriers and those without mutation (*P* = 0.049 and *P* = 0.033 respectively). Gender, tumour size, tumour localization, metastatic disease as well as urine and plasma epinephrine output were not different among the three carrier status groups.

**Figure 3:**
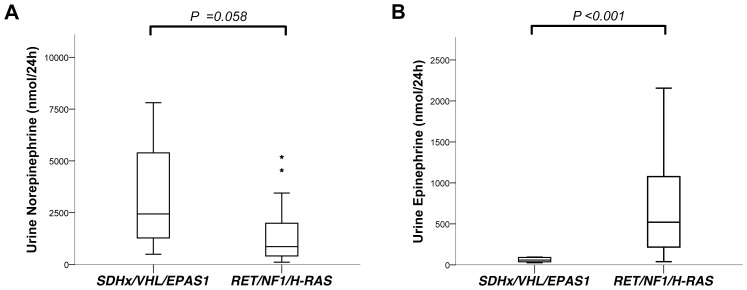
Box plots illustrating preoperative levels of urinary (A) norepinephrine and (B) epinephrine stratified accordingly to genotype clusters.

Stratification according to genotype into cluster 1; *SDHx*/*VHL*/*EPAS1* mutants and cluster 2; *RET*/*NF1*/*H-RAS* mutants, resulted in a difference in age at diagnosis between cluster 2 carriers (median 45) and patients without mutations (median 53, *P = *0.036). A borderline significance difference was also noted for PCC localization, there were one left adrenal and eight right adrenals affected in cluster 1 compared to eight left and eight right-sided tumours in cluster 2 (*P = *0.052). A difference in PCC lateralization was also noted between cluster 1 patients and those without discovered mutations (*P* = 0.028). In patients without mutation there were one case with multiple PGL and none bilateral PCC, different than in cluster 1 (4 cases total, *P*<0.001) and cluster 2 (5 cases total, *P = *0.004). Urine norepinephrine levels (Figures 3a and b) were higher in cluster 1 patients (median 2439/24 h, *P = 0.03*) and those without mutation (median 2181,5, *P = *0.018) compared to cluster 2 (median 862). Reversely Epinephrine levels were lower in cluster 1 (median 58/24 h, *P<0.001*) and in those without mutation (median 247 nmol/24 h, *P* = 0.002) compared to cluster 2 patients (median 520 nmol/24 h). Age at diagnosis, gender, plasma cathecolamines as well as recurrent and metastatic disease were not different among the three groups.

## Discussion

### Genetics

We have analysed 101 PCC and PGL tumours for SNVs in nine different genes, complemented by selective MLPA and SNP array analysis. A total of 33 patients (37%) had pathogenic variants in *SDHB*, *VHL*, *EPAS1*, *RET* and *H-RAS*. Including patients with clinical criteria of Neurofibromatosis type 1 and loss of heterozygosity at the *NF1* locus, 41% of the cohort could be associated with genetic aberrations in known genes. We did not find any pathogenic germline mutations that had not been previously discovered by clinical screening, this low frequency of germline mutations in apparently sporadic patients have previously been described in Swedish patients [40]. Considering the characteristics of the cohort with a predominance of benign and unilateral PCC having sporadic presentation, the frequencies of pathogenic genetic variants and patient carrier status were similar to those previously reported [8]. Loss of heterozygosity could be detected in tumour DNA from 11/12 patients having somatic or germline mutations in the *SDHB* or *VHL* genes. SNParray analysis of tumour DNA from patient 10 (VHL p.Arg161Gln) did not show LOH at any locus. This tumour sample will have to be carefully reviewed for contaminating wild type cells and re-analysed. Patients with clinical criteria of NF1 syndrome did not have a clinical diagnostic genetic test performed, probably due to the size of the *NF1* gene and the number of possible loci. Three of these patients tumours were analysed with SNParray that revealed loss of heterozygosity at the *NF1* loci in tumour DNA in all investigated cases. This strongly suggest that these patients do have a germline mutation in *NF1* [15], [18]. The interpretation of clinical correlations in sporadic patients presented by this study is limited by the absence of analysis of the *NF1* gene that was recently found to be commonly affected in patients with sporadic PCC and PGL [15], [18]. To analyse this extensive loci, further studies may utilize Next Generation Sequencing or SNP arrays [35], [41].

Multiplex ligation-dependent probe amplification analysis of constitutional DNA showed no copy number gains or losses in any of the investigated cases. Both the MLPA laboratory workflow and result analysis were performed using robust workflows by experienced clinical investigators, ensuring high reliability of these results.

### Variants of Unknown Significance

Multiple factors contribute to determine the disease causing impact of a specific genetic variant; variant deleteriousness, probands phenotype, family history, molecular and *in silico* characterization as wells as the allele frequency in a population without disease [42]. For SDHC Pro110Ser and Met164Leu, clinical presentation and family history did not indicate familial paraganglioma type 3. Analysis of the biochemical phenotype revealed a high norepinephrine to epinephrine ratio, pointing to a disease causing mutation in the *SDHx* or *VHL* loci [43]. Available literature did not support neither classification of pathogenic nor benign status of SDHC Pro110Ser and Met164Leu. *In silico* analysis determined these variants to have a benign effect on protein structure. But as *in silico* methods have a high rate of false negative predictions [44] and the fact that the alleles are not reported in any normal population we classified the variants as VUSs.

In patient 37 with the germline *VHL* p.Ser183Leu variant there was no family history suggesting VHL syndrome. The patient phenotype with a focal PGL did not strongly indicate a germline mutation even though up to 10% of patients may present with PGL [26]. The ratio of norepinephrine to epinephrine was high indicating a mutation in *SDHx* or *VHL* genes [43]. Screening of 190 healthy subjects did not find this variant nor was it found by a database search, supporting that the variant is not a common polymorphism. *In silico* calculation determined the variant as probable pathogenic. This variant’s contribution to patient disease was classified as unknown. However, several indicators point out this variant as potentially pathogenic and as most variants in the *VHL* gene are pathogenic, this variant should influence the clinical management of the proband.

### Genotype Clustering

The molecular phenotype of *EPAS1* mutated tumours has been disputed [16], [17]. However, clinical correlations and experimental investigations have indicated similarities of *EPAS1* lesions to *SDHx* and *VHL* mutated tumours. Catecholamine production detected in *EPAS1* carriers in this study suggested cluster 1 differentiation. The available literature strongly suggests a common pathway for *H-RAS* with cluster 2 genes *RET* and *NF1* [20], [43]. Additionally, the catecholamine output observed in *H-RAS* mutated tumours resemble that of *RET* and *NF1* [43]. We included *EPAS1* in cluster 1 and *H-RAS* in cluster 2 as well as patients with clinical criteria of NF1.

### Genotype Phenotype Correlations

As previously described, germline carriers were significantly younger at time of diagnosis, and had a higher frequency of multifocal disease [26]. Among carriers with somatic mutations there were no recurrences, nor were there any cases of metastatic disease. Stratified into cluster 1 and 2 genotypes, differences in biochemistry output was observed, in line with previous studies investigating germline carriers [43]. A difference in the location of adrenal tumours was noted with cluster 1 patients having the right adrenal affected in all but one case. No obvious explanation to this lateralisation was found in the literature and studies of larger cohorts is needed to confirm this observation.

Five of 13 patients with recurrent disease were carriers of germline mutations in *SDHB* or *RET,* the remaining eight patients had sporadic disease presentation. The frequency of discovered variants in included loci were different in metastatic (22%) compared to non-metastatic cases (40%). Further genetic investigations of sporadic patients with recurrent and/or metastatic tumours could help to identify novel disease causing genes associated with a high risk of recurrence. Genetic prognostic markers could potentially be of value in a clinical context in order to personalize the extent of follow up [5], [45]. Planned studies aiming at identifying factors that might predict recurrent disease would favourably include tumour genotype characterization.

### Tumour Genotype in Clinical Management

There is a growing rationale for analysing somatic events in PCC and PGL tumours as a diagnostic test: (1) *EPAS1* mutations may occur early in embryogenesis and these mosaic carriers are not found by analysis of DNA in peripheral blood [16], [46]; (2) Translational studies have suggested using genotype as predictive markers for sensitivity to targeted therapy; *SDHx*/*VHL* mutations might benefit from antiangiogenic treatment whereas *RET*/*NF1*/*TMEM127*/*MAX* driven tumours could benefit from inhibitions of kinase pathways [47], [48]. The present study further suggests that tumour genotype might have additional prognostic implications. Even though methods for using formalin fixed archived tissue are evolving rapidly, analysis of tumour DNA is favourably performed using high quality fresh frozen genetic material thus discussions regarding the current routines, archiving only formalin fixed tissue, are warranted.

## Conclusion

Somatic mutations are frequent events in PCC and PGL tumours. In patients with somatic carrier status there were no cases of recurrent nor metastatic disease. These findings suggest that analysis of tumour DNA could have an impact on the management of PCC and PGL patients and should be further investigated as prognostic factors in these diseases.
